# Can local adaptation research in plants inform selection of native plant materials? An analysis of experimental methodologies

**DOI:** 10.1111/eva.12379

**Published:** 2016-10-04

**Authors:** Alexis L. Gibson, Erin K. Espeland, Viktoria Wagner, Cara R. Nelson

**Affiliations:** ^1^ College of Forestry and ConservationUniversity of MontanaMissoulaMTUSA; ^2^ Pest Management Research UnitUSDA‐ARS NPARLSidneyMTUSA; ^3^ Present address: Department of Botany and ZoologyMasaryk UniversityKotlářská 2CZ‐611 37BrnoCzech Republic

**Keywords:** ecological experiments, experimental design, experimental methodology, lifetime fitness, local adaptation, plants

## Abstract

Local adaptation is used as a criterion to select plant materials that will display high fitness in new environments. A large body of research has explored local adaptation in plants, however, to what extent findings can inform management decisions has not been formally evaluated. We assessed local adaptation literature for six key experimental methodologies that have the greatest effect on the application of research to selecting plant materials for natural resource management: experimental environment, response variables, maternal effects, intraspecific variation, selective agents, and spatial and temporal variability. We found that less than half of experiments used reciprocal transplants or natural field conditions, which are both informative for revegetation and restoration. Population growth rate was rarely (5%) assessed, and most studies measured only single generations (96%) and ran for less than a year. Emergence and establishment are limiting factors in successful revegetation and restoration, but the majority of studies measured later life‐history stages (66%). Additionally, most studies included limited replication at the population and habitat levels and tested response to single abiotic selective factors (66%). Local adaptation research should be cautiously applied to management; future research could use alternative methodologies to allow managers to directly apply findings.

## Introduction

Local adaptation is the process by which resident genotypes exhibit higher fitness in their home environment compared with nonlocal genotypes due to divergent selection as a consequence of variation in environment (Kawecki and Ebert 2004). Over the course of the 20th century, research on local adaptation has expanded from a primary focus on long‐term evolutionary processes, such as speciation (Jordan 1905), to a broader set of issues including rapid evolutionary processes and responses to changing environmental conditions (Barrett et al. 2008; Leger and Espeland 2010; Hoffmann and Sgro 2011).

Meanwhile, scientists and managers are increasingly using results of local adaptation research (LAR) to inform complex management decisions (Hufford and Mazer 2003), such as assisted migration for climate change mitigation (Vitt et al. 2010), and choice of native plant materials for revegetation and restoration (McKay et al. 2005). For example, positive findings of adaptation to local selective pressures (Joshi et al. 2001; Leimu and Fischer 2008; Hereford 2009) have been used as an argument in favor of primarily using local ecotypes in restoration (USDI and USDA 2002; Johnson et al. 2010a; Vander Mijnsbrugge et al. 2010b). Native plant material choices impact the viability and adaptive potential of restored populations (Williams 2001; Broadhurst et al. 2006; Aavik et al. 2012), as well as the feasibility of using locally collected seeds in large‐scale restoration (Merritt and Dixon 2011). Because of this, it is critical to understand the extent to which LAR can be broadly applied to land management.

Findings of LAR have substantially advanced our understanding of local adaptation in plants, yet it remains unclear to what extent the methods used in previous LAR allow us to assess the magnitude of local adaptation at a scale relevant to land management. Three issues could complicate the application of LAR to management questions. (i) Although LAR aims to understand adaptation at the metapopulation level, the number of populations and habitats sampled is typically small; (ii) the ability to apply findings to restoration at the landscape scale depends in large part on whether experimental methodologies capture selective pressures at relevant temporal and spatial scales; (iii) in addition, the experimental environment, response variables selected and maternal effects all affect the extent to which one can apply LAR to native plant material choices. Given these issues, we conducted a literature review to assess to what extent the methodology of LAR can be extrapolated to inform land managers about the choice of best native plant material for restoration. Specifically, we assessed six experimental methodologies:

*Experimental environment—*The most conclusive method for detecting local adaptation is through replicated reciprocal transplant experiments that compare fitness in multiple home and foreign sites (Kawecki and Ebert 2004; Blanquart et al. 2013). Multiple sites allow researchers to identify traits related to fitness that have been selected by the environment. LAR will be informative for land management if experiments use whole environments (Nuismer and Gandon 2008), and occur at multiple sites and in experimental conditions that are similar to those found during revegetation. Under this scenario, researchers would gain insight into the scale of population differentiation and the frequency of local adaptation. By contrast, experiments conducted at single sites, such as common garden studies, can only show phenotypic variation among populations, not whether fitness is higher for local versus nonlocal populations. Common garden or greenhouse studies could help identify which population will perform best at a specific revegetation site, but in the majority of cases, native plant materials will be used at multiple sites with unknown conditions. Additionally, common garden and reciprocal transplant studies can be used in conjunction with gaining deeper insight into the drivers of local adaptation (Nuismer and Gandon 2008), but the usage of unaltered field environments is especially important to accurately assess fitness when local adaptation is only observed under specific environmental conditions (cryptic adaptation), such as the presence of native plant community competitors (Knight and Miller 2004; Bischoff et al. 2006; Rice and Knapp 2008).
*Measures of response—*From a restoration perspective, population growth rate is the most relevant direct fitness measure because it indicates long‐term population viability (Menges 1990; Rice and Emery 2003). Unlike individual trait measurements, such as biomass or reproductive success, multiplicative population growth rate incorporates multiple parameters related to population persistence and growth. One common metric for assessing population growth rate is lambda (*λ*), the proportional change in population size from one generation to the next; simply put, *λ* must be ≥1 for a population to persist. Plant traits that respond to selection in the populations' home sites can be also used to detect evidence of local adaptation, but they are less likely to be directly related to fitness and may not show a signal for response to selection. Furthermore, ecological restoration benefits from research conducted across multiple life‐history stages and generations, as fitness responses can vary across these scales (Donovan and Ehleringer 1992; Kelly 1992; Rice and Knapp 2008). Given that the majority of revegetation projects rely on seeds to establish native plants (Koch 2007; Broadhurst et al. 2008), research that focuses the expression and magnitude of local adaptation during germination and establishment may provide especially important information for land management.
*Maternal effects—*Observed phenotypic differences among populations can result from differences among genotypes (local adaptation) or maternal effects (Roach and Wulff 1987). Adaptive maternal effects have been found to increase performance of the progeny of maternal plants exposed to drought (Sultan et al. 2009), herbivory (Agrawal 2001, 2002), herbicide (Bozorgipour and Snape 1997) and shading (Donohue and Schmitt 1999; Galloway and Etterson 2007; Bell and Galloway 2008) in these environments. In addition, the effects on phenotype of progeny can persist for multiple generations (Miao et al. 1991). For populations that remain in place in the landscape, maternal effects may make fitness in sympatry even stronger (Espeland and Rice 2012). In the case of land management, however, seeds are moved away from the maternal plant environment and expected to show the same traits and performance. Maternal effects will not mask local adaptation when it is present, but they may be confused with local adaption (when it is absent) or inflate the observed magnitude of fitness differences (when it is present). When maternal effects drive adaptive plant traits and when maternal environments (i.e. seed production farms) differ from target environments, determining whether traits are the result of maternal effects or local adaption will be critical for predicting seed and plant performance in revegetation.
*Number of populations and habitats—*Assessing the spatial scale of environmental and genetic differentiation requires sampling many individuals and populations (Manel et al. 2003), especially if there is significant variation among populations. Just as populations differ in the selective pressures they experience, they also differ in the magnitude and direction of response to those pressures (Thompson et al. 2002; Leger and Espeland 2010), and populations may show fitness differences unrelated to local adaptation due to habitat quality or genetic factors such as inbreeding (Blanquart et al. 2013). These issues combine to make it difficult to determine which selective factors are important drivers of adaptive trait differentiation and the scale over which they operate. Additionally, the type and number of habitats sampled from influences the scale at which local adaptation can be assessed. When planning a revegetation project, the practitioner calculates the likelihood of differential genotypic success in the environment; using multiple populations collected from many habitats in LAR enhances the ability of practitioners to make these difficult decisions by clearly defining the magnitude, scale and drivers of local adaptation. The popularity of genecological studies that measure hundreds of field‐collected populations in common gardens to generate geographic limits of appropriate seed transfer (e.g. Johnson et al. 2010b; St Clair et al. 2013) is evidence that this magnitude of population sampling may be necessary to assist practitioners in seed selection.
*Selective agents—*Understanding the factors that drive population differentiation is important in choosing native plant materials. Plant species can be adapted to both abiotic conditions (e.g. soil and climate; Macel et al. 2007; Goransson et al. 2009) and biotic factors (e.g. pollinators and soil pathogens; Svenning et al. 1991; Thrall et al. 2002; Streisfeld and Kohn 2007), and interactions between factors can alter the observance or strength of local adaptation (Hufford et al. 2008; Lau et al. 2008). Understanding the impact of multiple selective factors on population fitness will not only help managers identify which factors define ‘local’, but also provides information about the field conditions under which higher home‐site fitness is observed. As ecological restoration and land management are carried out in the realm of communities and ecosystems, research needs to take a multitude of selective factors and their interactions into account.
*Environmental variability—*Beyond biotic and abiotic factors that are largely consistent across years, factors that vary across time can also be important agents of selection. For example, selective agents that drive local adaptation may only act on some generations of the target species (Rice and Mack 1991; Geber and Griffen 2003; Thompson et al. 2007) and impacts on nonlocal sources may not be apparent for decades (Millar and Libby 1989). Spatial variation is often used in ecological experiments to predict what would occur over a longer time span (Haubensak and Parker 2004) because temporally rare events required for the expression of local adaptation*—*such as disease or drought*—*are more likely to be captured when multiple sites are used. Therefore, the number of environments and the type of variation encompassed within LAR (either by conducting an experiment over multiple experiment years or using many sites) is important for assessing the constancy of the expression of local adaptation and the comparative risk of using nonlocal genotypes.

To date, reviews of LAR have focused on identifying the overall frequency and drivers of local adaptation (Leimu and Fischer 2008; Hereford 2009, 2010) or on best practices for researching local adaptation (Kawecki and Ebert 2004; Kawecki et al. 2012; Blanquart et al. 2013). There is an additional need to assess the extent to which existing LAR can inform decisions regarding genetically appropriate plant materials for land management; these decisions require an understanding of how selection across the landscape shapes plant traits that are most important for restoration establishment and long‐term success. In addition to genetic diversity in quantitative trait loci, local adaptation is an important consideration for successful revegetation, and policy and practice are increasingly focusing on using it to select where to collect and move plant materials. We conducted a literature review in order to quantify to what extent LAR has integrated six key methodological considerations and can guide choices of native plant materials for management.

## Materials and methods

We performed a literature search in ISI Web of Science using the search terms ‘local adapt*’ and ‘plant*’, for the period of 1965 to February 2013. A total of 1046 studies were identified. We reviewed titles, abstracts and keywords of each article to determine suitability for inclusion and excluded studies that did not focus on local adaptation in vascular plants (439 studies), had primary species of interest that were non‐native invasive species (113 studies), used only molecular analysis (93 studies), focused on crop plant(s) (42 studies) or were not experimental (e.g. theoretical, modeling and review papers; 124 studies). If a study was comprised of multiple experiments, we recorded data on each experiment individually. The final analysis comprised 234 articles describing 308 experiments. The experiments tested for local adaptation in 278 different plant species, mostly forbs (69%) and graminoids (20%) and, to a lesser degree, trees (9%) and shrubs (2%). Of the nontree species, 74% were perennial and 26% were annual.

For each experiment, we assessed six methodological variables that are relevant for ecological restoration: experimental environment, measures of response, maternal effects, among‐population variability, selective agents, and spatial and temporal variability. We recorded components of the experimental environment (type of experiment, site type, inclusion of the home plant community) as well as the response variables analyzed (the life stages studied, whether data were collected over the plant's entire lifespan, and whether multiple generations were studied). To classify the extent to which experiments controlled maternal effects, we recorded whether plant materials used in each study were the result of collections from a controlled environment, or if authors accounted for maternal effects using early‐stage measurements (initial seed weight or initial plant size) as covariates in statistical analysis; these methods are commonly accepted and utilized to control for maternal effects as seed weight and plant size can be indicative of maternal provisioning. We also recorded the number of different habitat types that populations were collected from as reported by authors (e.g. grassland and dune sites, inland and coastal sites) and the number of different populations from which plant material was collected (defined by authors). We identified the type and number of agents of natural selection that were tested within each experiment (biotic interactions and abiotic factors). To determine the spatial and temporal variability captured in experimental design, we recorded the duration of each experiment (rounded to the nearest year), the number of environments that were used in studies that were done in unmanipulated field conditions, or the number of experimental conditions tested whether investigators used treatments to create multiple experimental environments.

## Results

### Experimental environment

Thirty‐nine percent of experiments used reciprocal field transplants among the populations' home sites, whereas 33% used common garden designs (Table 1). Roughly half (*N* = 55) of the common garden experiments were conducted at a single site. Greenhouse and growth chamber experiments were the least frequently used (28%, *N* = 87). Approximately equal numbers of experiments were performed in natural sites (41%, *N* = 125) as in artificial settings (pots, greenhouses and growth chambers; Table 1). Sixty‐eight percent of experiments (*N* = 208; Table 1) removed local vegetation from the experimental environment.

**Table 1:** Frequency (number and %) of use of six key experimental methodologies in local adaptation experiments (*N *=* *308)

Variable	Frequency	
	No.	%
Experimental environment		
Experiment type		
Reciprocal transplant	120	39
Common garden	101	33
Greenhouse	87	28
Site type		
Natural site	125	41
Artificial conditions	133	59
Other vegetation included		
Only target plant species present	208	68
Native vegetation intact or added	78	25
Measure of response		
Fitness		
Population growth rate (*λ*)	14	5
Reproductive success	137	44
Germination/emergence	63	20
Survival/mortality	126	41
Damage by herbivores/pathogens	22	7
Visitation from mutualists	3	1
Size (e.g. biomass, number of leaves, circumference)	182	59
Other	46	15
Life stages		
Germination	79	26
Juvenile	258	84
Reproduction	173	56
2 stages	124	40
All 3 stages	41	13
Multiple generations		
Yes	12	4
No	296	96
Entire life cycle		
Yes	64	21
No	244	79
Number of populations and habitats		
Number of populations (mean)	8	–
Number of habitats plant material collected from (mean)	3	–
Maternal effects		
Plant material from controlled environment	89	29
Weighed seeds	37	12
Kept maternal families separate	50	16
Initial plant size used as covariate	51	17
Selective agents		
Biotic factors		
Plant	40	13
Herbivore	20	6
Pathogen	3	1
Mutualist	7	2
Soil biota	13	4
Multiple biotic factors	5	2
Biotic and abiotic factors	42	14
Abiotic factors		
Climate	144	47
Soil	65	21
Light	10	3
Disturbance	31	10
Distance	3	1
Other	40	13
Multiple abiotic factors	27	9
Environmental variability		
Length of experiment (years; mean)	2	–
Number of sites or created environments (mean)	4	–

### Response variables

**Figure 1:**
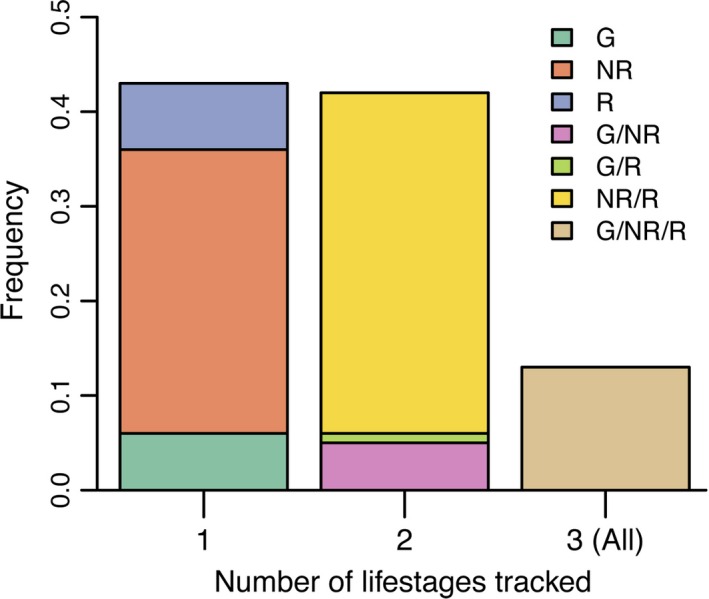
Frequency of local adaptation experiments (proportion; *N *=* *308) that tracked plants during germination (G), nonreproductive juvenile or adult (NR), and reproductive (R) life stages, or combinations thereof.

Although 82% of experiments calculated a measure of fitness, only 5% (*N* = 14) included *λ* as a response variable (Table 1). Biomass was the most frequently used measure of fitness (59%), followed by reproductive success (44%). The most common life‐history stage assessed was nonreproductive, followed by reproductive adult (Table 1); germination was the least commonly tracked (26%; Table 1). Forty‐one percent of experiments tracked two life stages, and 13% tracked plants across all three life stages (Fig. 1). The majority of studies did not follow plants until death (77%, *N* = 244; Table 1) or track multiple generations (96%, *N* = 296; Table 1).

### Maternal effects

Approximately three quarters of experiments controlled for maternal effects in some way. However, most of these (45%, *N* = 138) used initial plant size or seed as a statistical covariate, or kept maternal families separate in statistical analysis (Table 1). Only a third (29%) included plant material that had been grown in a controlled maternal environment.

### Among‐population variability

**Figure 2:**
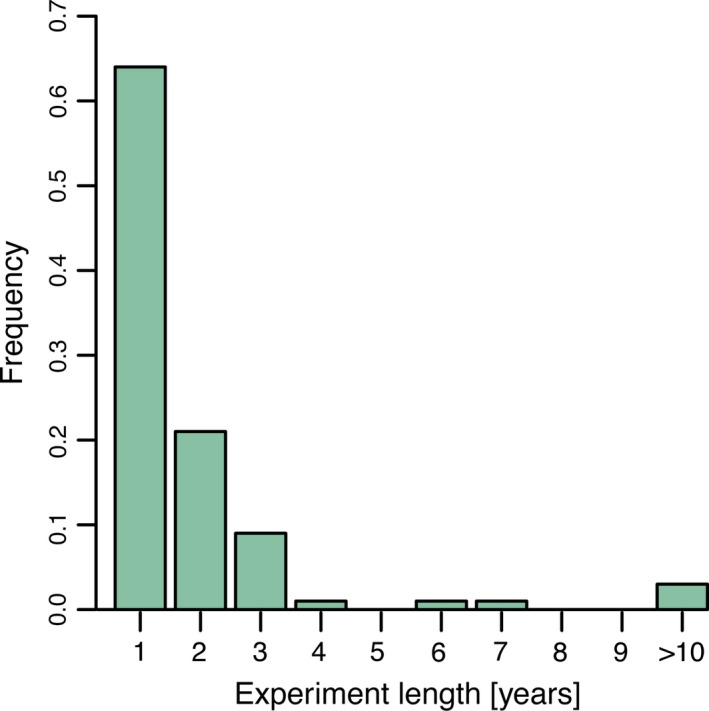
Frequency of local adaptation experiments (proportion, *N *=* *308) by level of replication (none to >10) for populations (black bars) and habitats (white bars). Population was defined by authors as a single source of plant materials. Habitat refers to areas from which populations were collected.

We found wide variation in the number of collection populations and habitats (Fig. 2). On average, experiments used plant materials collected from eight populations and three different habitat types.

### Selective agents

The majority of experiments tested adaptation to abiotic factors (89%, *N* = 271). Biotic factors were rarely considered (25%, *N* = 76), and only 2% (*N* = 7) assessed adaptation in the presence of multiple biotic factors (Table 1). The majority of studies that tested abiotic factors focused on climate (Table 1). Additional factors were overall ecological and geographic differences between populations, salt‐spray tolerance and inundation gradients (Table 1). Ten percent (*N* = 27) of studies tested adaptation to multiple abiotic factors or abiotic and biotic factors in combination (14%, *N* = 42).

### Spatial and temporal variability

**Figure 3:**
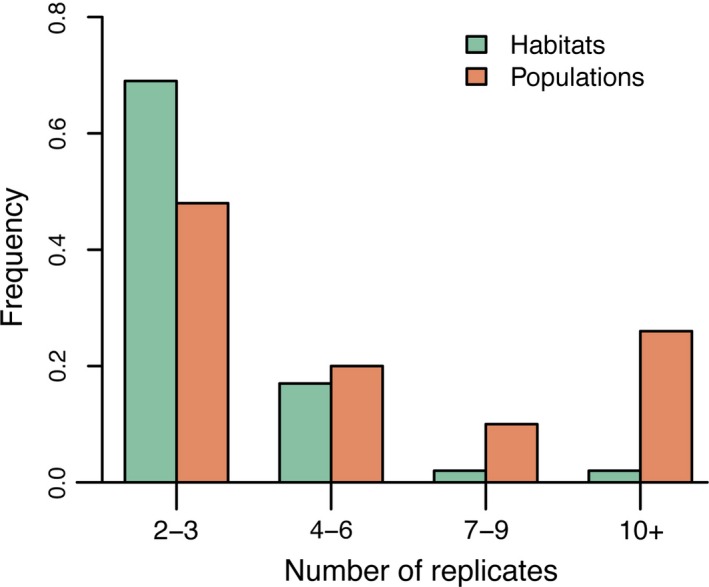
Frequency of local adaptation experiments (proportion; *N *=* *308) by experimental duration in years.

On average, experiments ran for 2 years, with the median being <1 year (Fig. 3). The longest running experiment lasted 45 years (Gomory et al. 2012). There was a wide range of variability in the number of environments experiments occurred in (sites or environmental conditions if a greenhouse or common garden study; mean = 4, median = 3; Table 1).

## Discussion

Practitioners have increasingly used results from LAR to guide management decisions (e.g. Vander Mijnsbrugge et al. 2010a). However, our results suggest that findings from LAR are not easily transferable to land management due to experimental constraints. In particular, LAR primarily used experimental environments that did not mimic natural conditions, chose response variables that did not reflect lifetime fitness, excluded biotic and multiple selective factors, and used limited replication and experimental duration. While these methodological choices do not reflect the quality or findings of individual experiments designed to test specific factors of interest, practitioners should interpret results from LAR with caution.

As with previous reviews (Leimu and Fischer 2008; Hereford 2009), we found that only a portion of LAR directly addresses local adaptation through the use of reciprocal transplant studies (39%). The frequencies of finding local adaptation do not appear to differ between common garden and reciprocal transplant studies (Leimu and Fischer 2008), but observed differences among populations can differ from common garden studies in strength (Table 2). Perhaps more importantly for applying LAR, less than half of studies occurred in natural environments (41%), or retained the native plant community (25%). Although removing confounding factors such as natural site variation and the home plant community can make it easier to study factors of interest, it impacts both probably of detection and whether findings are relevant *in situ* (McCarragher et al. 2011; Ehlers et al. 2012; Pankova et al. 2014; Table 2). Furthermore, the choice of traits or inclusion of *λ* in LAR is relevant for whether findings of higher fitness translate to increased population persistence, and the two may give contradictory results that alter whether local adaptation is observed (Table 2). We found that only 40% of LAR used direct fitness measures (either survival or reproductive success) and very few (5%) used *λ*. Incorporating multiple life stages increases the applicability of LAR to management as the use of local native plant materials is often predicated on the assumption that local adaptation will increase population fitness at critical life stages, yet local populations may not show consistent trends of higher fitness across their entire life cycle (Table 2). Germination and emergence are often the limiting factors in revegetation success (Khurana and Singh 2001; Pywell et al. 2003) and critical to population regulation (Horvitz and Schemske 1995; Freville and Silvertown 2005); however, less than a quarter of experiments incorporated these life stages as a measure of fitness.

**Table 2:** Examples of local adaptation experiments that incorporated variables that are informative to ecological restoration, and a brief summary of the impact of the variable on the findings of local adaptation or population differentiation. Papers did not incorporate all six variables equally, and summary findings could be influenced by the remaining five variables

Variable	Authors	Summary
*Reciprocal versus common garden*	Raabova et al. (2011)	Results from reciprocal transplant and common garden experiments differed in the observed level of population differentiation. While both types of experiments showed greater height of local versus foreign plants, there were smaller differences in height in the field compared to the common garden. This indicates that the magnitude of difference was smaller in the reciprocal transplant compared to common garden experiment
*Inclusion of native vegetation*	Bischoff et al. (2006)	Inclusion or exclusion of the local plant community altered the detection and magnitude of local adaptation in two species. Fitness was higher for *Plantago lanceolata* when the native plant community was present, while *Holcus lanatus* showed lower home‐site fitness with the local plant community present
*Population growth rate (λ)*	Becker et al. (2006)	Findings about population fitness were different when fitness in traits and lifetime fitness (*λ*) were assessed. Four of six life‐history traits studied showed nonsignificant differences between home versus away populations; however, *λ* showed a significant home‐site advantage
*Multiple life stages*	Raabova, Muenzbergova and Fischer (2007)	Findings of local adaptation depended on life stage assessed. Evidence of local adaptation was seen in the number of germinates (up to 68% higher in local versus foreign populations), but no consistent evidence of local adaptation was found in adults
*Multiple populations/habitats*	Hereford and Winn (2008)	Evidence of home‐site advantage was rare and depended on the degree of habitat similarity. Local adaptation was not found when populations were from the same habitat type, but was significantly likely to be found when populations were from different habitats
*Plant materials from controlled environment*	Bischoff and Muller‐Scharer (2010)	Maternal effects impacted level of population differentiation detected and observed traits. Populations showed less differentiation when using plants from controlled crosses than parent plants. The ranking of populations in the F1 generation also changed for some traits. Maternal effects were independent of seed mass
*Multiple factors*	Lau (2006)	Findings of adaptation varied when multiple biotic factors versus a single factor were studied. When grown only with the invasive *Medicago polymorpha*,* Lotus wrangelianus* plants from invaded sites showed adaptation to invasion. There was no evidence of adaptation to the invader when the insect herbivore *Hypera brunneipennis* was included
*Experimental length*	Bennington et al. (2012)	Experimental length was important for the observation and magnitude of local adaptation. For *Dryas octopetala*, the strength of local adaptation increased over a decade. For *Eriophorum vaginatum*, there was no evidence of local adaptation until 17 years after transplant

Reciprocal transplants and direct fitness measures are just two of the important experimental considerations for applying LAR to restoration; given the expense of using local seeds, managers need to be confident that local sources will result in long‐term increased fitness in restored populations. Replication over space and time and the inclusion of relevant selective agents are equally important, but rarely adequately addressed. Thus, it is unknown whether findings of local adaptation are due to fitness differences in response to selective agents or trait differentiation unrelated to fitness, and it could be additionally difficult to determine whether local seeds will be consistent in showing higher fitness under altered site conditions (Table 2). The limited number of habitats plant materials was collected from increases the risk that LAR has selectively used populations from a few highly contrasting environments, thereby increasing the chance of finding fitness differences regardless of experimental methodology used (Hereford and Winn 2008; Hereford 2009; Table 2), directly limiting the application of LAR to decisions regarding the scale and importance of local adaptation in choosing plant materials. In addition, Siepielski et al. (2009) found that the strength, direction and sources of selection frequently change among years (but see Morrissey and Hadfield 2012)*—*the short duration and limited testing conditions of most LAR indicate that even normal variation at experimental sites is unlikely to be captured. The magnitude fitness differences due to local adaptation can change over decades (Table 2), leaving the question of whether short duration research accurately represents the population dynamics that will occur postrevegetation.

One essential consideration that was frequently addressed in LAR was maternal effects. Although Hereford (2009) anecdotally noted that most LAR experiments did not account for them, we found that 74% of experiments controlled for maternal effects in some way, although only 29% used plant materials from common environments. Maternal effects can increase the observed differences among populations (Table 2) and could alter the interpretation of higher fitness. The frequent use of measures to control for maternal effects suggests that most LAR does not confound transgenerational plasticity and genetic differentiation. In this aspect, LAR can be appropriately applied to problems of moving genotypes from one environment to another.

### Future direction

The difficulty of conducting LAR that can be applied to management may in part stem from logistical obstacles in research and dependence on short‐term funding. For instance, the inclusion of *λ* as a response variable is complicated by that fact that: (i) extended periods of data collection are required to accurately estimate it for long‐lived species (Che‐Castaldo and Inouye 2011); and (ii) that estimates of *λ* in plants require accounting for factors such as seed banks (Adams et al. 2005), dormancy (Miller et al. 2012) and nonseed reproduction (Nault and Gagnon 1993). It can also be difficult to study multiple selective factors in concert or to determine which selective agents are important in natural field settings.

**Figure 4:**
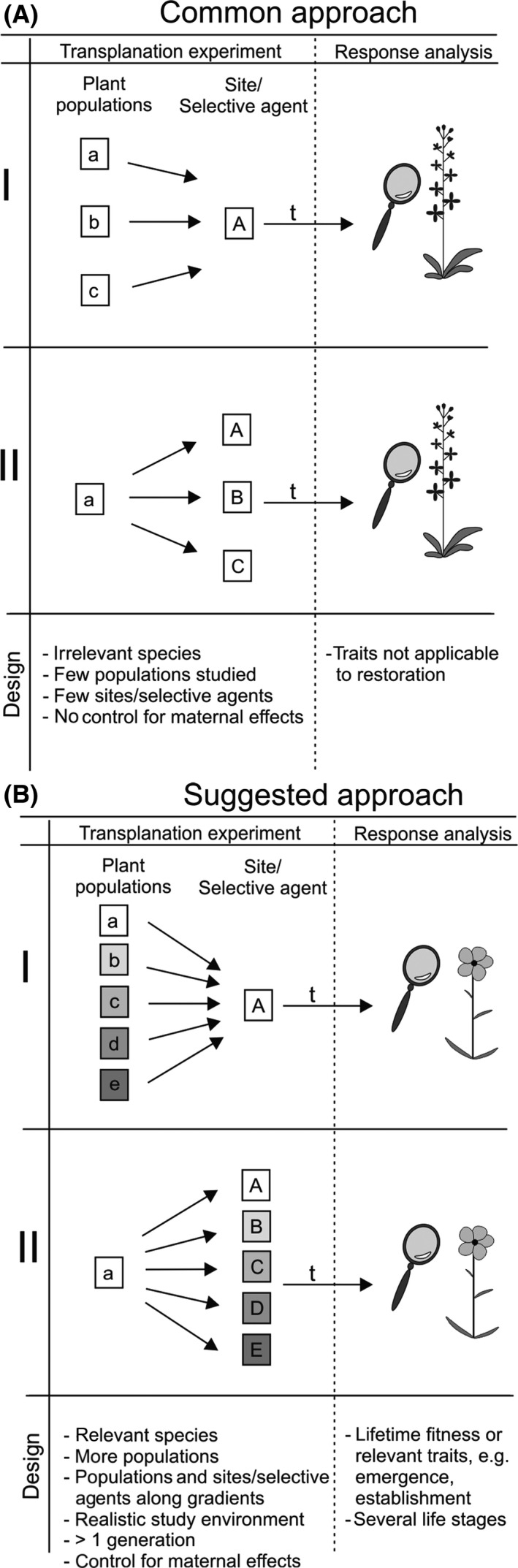
Schematic graph of the (A) common approach to local adaptation experiments and (B) a suggested approach that could make experiments more relevant to land management. Small letters (a–e) indicate plant populations; capital letters (A–E) indicate sites or selective agents; and t indicates time after the beginning of the experiment. In (B), gray‐shaded colors, underlying boxes (a–e, A–E) represent an environmental or geographic gradient. In panel a, material from multiple plant populations is crossed either at one site (I) or with one selective agent (II). In panel b, material from multiple plant populations is crossed with multiple sites or selective agents. Dots indicate that reciprocal transplant is replicated at the remaining sites.

Even though ideal experimental considerations are likely unattainable, investigators interested in research for restoration application could address a greater set of considerations in their designs (Fig. 4). First, they could increase the number of populations and the sites and life‐history stages assessed, and increase study duration. Second, if utilizing *λ* is not feasible, researchers could test for fitness differences in response to selective agents at specific life‐history stages concurrently, rather than sequentially. Third, performing LAR over environmental gradients or clines (Etterson 2004; Fant et al. 2008) has the advantage of determining the importance of landscape variability over multiple scales on the expression of local adaptation. Researchers could increase their participation in inter‐regional or intercontinental collaboration to allow the inclusion of more populations and habitats in local adaptation experiments. Alternatively, researchers and managers could increase their collaboration by tracking the success of locally collected seeds at restoration sites. Finally, combining reciprocal transplants in natural conditions with controlled common garden experiments could provide greater information about the drivers and magnitude of local adaptation (Raabova et al. 2011). These suggestions are valid for all LAR and would help researchers adhere to best practice. Results from experiments that included these six factors illustrate their importance in assessing local adaptation, and managers should consider how directly LAR could inform policy.

## Data accessibility

Data for this study were collected from peer‐reviewed published literature.
